# A polymorphism in the enhancer region of the thymidylate synthase promoter influences the survival of colorectal cancer patients treated with 5-fluorouracil

**DOI:** 10.1054/bjoc.2001.2007

**Published:** 2001-09

**Authors:** B Iacopetta, F Grieu, D Joseph, H Elsaleh

**Affiliations:** Department of Surgery, University of Western Australia, Nedlands, 6907, Australia; Department of Radiation Oncology, Sir Charles Gairdner Hospital, Nedlands, 6009, Australia; Department of Medicine, University of Western Australia, Nedlands, 6907, Australia

**Keywords:** colorectal cancer, adjuvant chemotherapy, thymidylate synthase, polymorphism

## Abstract

High levels of thymidylate synthase (TS) expression have been associated with poor survival of colorectal cancer (CRC) patients to 5-fluorouracil (5-FU)-based chemotherapy. Recent evidence suggests that a polymorphism within the enhancer region of the TS gene promoter can influence TS expression, with the triple repeat homozygote (3R/3R) being associated with significantly higher tumour TS levels than either the double repeat homozygote (2R/2R) or heterozygotes (2R/3R). In the present study we investigated whether TS genotype was associated with the degree of survival benefit from chemotherapy in 221 Dukes' C stage CRC patients. Patients with the 3R/3R polymorphism (*n* = 58, 26%) showed no significant long-term survival benefit from chemotherapy (RR = 0.62, 95% CI: 0.30–1.25, *P* = 0.18), whereas those with the 2R/2R or 2R/3R genotype (*n* = 163, 74%) showed significant gains in survival from this treatment (RR = 0.52, 95% CI: 0.52–0.82, *P* = 0.005). These results demonstrate that a polymorphism within the TS gene, probably through its effect on TS expression levels, can influence the survival benefit obtained by CRC patients from 5-FU-based chemotherapy. © 2001 Cancer Research Campaignhttp://www.bjcancer.com


*British Journal of Cancer* (2001) **85**(6), 827–830
© 2001 Cancer Research Campaign
doi: 10.1054/ bjoc.2001.2007, available online at http://www.idealibrary.com on IDEAL

### A polymorphism in the enhancer region of the thymidylate synthase promoter influences the survival of colorectal cancer patients treated with 5-fluorouracil

**B Iacopetta[1], F Grieu[2], D Joseph[2,3] and H Elsaleh[1,2]**

[1]Department of Surgery, University of Western Australia, Nedlands 6907, Australia; [2]Department of Radiation Oncology, Sir Charles Gairdner Hospital, Nedlands 6009, Australia; [3]Department of Medicine, University of Western Australia, Nedlands 6907, Australia

**Summary** High levels of thymidylate synthase (TS) expression have been associated with poor survival of colorectal cancer (CRC) patients to 5-fluorouracil (5-FU)-based chemotherapy. Recent evidence suggests that a polymorphism within the enhancer region of the TS gene promoter can influence TS expression, with the triple repeat homozygote (3R/3R) being associated with significantly higher tumour TS levels than either the double repeat homozygote (2R/2R) or heterozygotes (2R/3R). In the present study we investigated whether TS genotype was associated with the degree of survival benefit from chemotherapy in 221 Dukes' C stage CRC patients. Patients with the 3R/3R polymorphism ($n$ = 58, 26%) showed no significant long-term survival benefit from chemotherapy (RR = 0.62, 95% CI: 0.30–1.25, $P$ = 0.18), whereas those with the 2R/2R or 2R/3R genotype ($n$ = 163, 74%) showed significant gains in survival from this treatment (RR = 0.52, 95% CI: 0.52–0.82, $P$ = 0.005). These results demonstrate that a polymorphism within the TS gene, probably through its effect on TS expression levels, can influence the survival benefit obtained by CRC patients from 5-FU-based chemotherapy. © 2001 Cancer Research Campaign http://www.bjcancer.com

**Keywords**: colorectal cancer; adjuvant chemotherapy; thymidylate synthase; polymorphism

Clinical trials have shown that a small proportion of Dukes' C stage CRC patients derive a survival benefit from adjuvant chemotherapy using 5-FU-based regimes (Moertel, 1994). Predictive factors for the efficacy of this treatment include the presence of tumour-specific genetic alterations such as *TP53* mutation (Ahnen et al, 1998) and the microsatellite instability (MSI) phenotype (Elsaleh et al, 2000). In addition to somatic alterations, genotype-related differences in the level of expression and function of various genes involved in the response to 5-FU may also have an impact upon the efficacy of treatment. Identification of both the somatic and germ-line factors that influence tumour cell response to 5-FU would greatly improve the selection of CRC patients to receive this form of treatment. Good predictive markers would be particularly useful for Dukes' B stage CRC where the benefits of adjuvant chemotherapy remain controversial (Anonymous, 1999; Mamounas et al, 1999).

The TS enzyme catalyses conversion of dUMP to dTMP, the latter being the sole source of thymidine nucleotides necessary for DNA synthesis. Inhibition of TS activity is thought to be one of the major mechanisms for the antitumoural properties of 5-FU (Santi et al, 1974). The cytotoxic effect of this drug occurs through the action of the 5-FU metabolite, FdUMP, which competes with dUMP for a binding site on the TS protein. Numerous in vitro (Johnston et al, 1992; Beck et al, 1994; van Triest et al, 1999) and clinical studies (Peters et al, 1994; Lenz et al, 1996; Leichman et al, 1997; Salonga et al, 2000) have demonstrated a correlation between TS levels and response to 5-FU, with high levels generally being associated with a poor response. Identification of the factors that regulate TS expression would therefore be of considerable benefit in understanding the mechanisms of tumour cell resistance to 5-FU.

Recent studies on the enhancer region of the TS promoter have identified a 28-bp double or triple tandem repeat polymorphism (Horie et al, 1995) that has been associated with the level of TS expression both in vitro (Horie et al, 1995) and in clinical tumour specimens (Kawakami et al, 1999). Triple repeat homozygotes (3R/3R) are associated with a 2–4-fold higher TS activity level compared to double repeat homozygotes (2R/2R) or heterozygotes (2R/3R). Therefore it might be expected that the TS polymorphism, through its influence on TS activity level, could influence the clinical outcome following treatment with 5-FU. In the present study we have examined the survival benefit from 5-FU in a large series of Dukes' C CRC patients that were stratified according to TS genotype.

### METHODS

### Tumour specimens

Tumours analysed in this study were from a larger series of Dukes' C CRC described recently by our laboratory (Elsaleh et al, 2000). A total of 221 cases were studied, of which 117 received standard, 6 monthly cycles of 5-FU-based adjuvant chemotherapy. Because of a bias towards the selection of younger patients to receive chemotherapy, an upper age limit of 70 years was set for those in the non-chemotherapy group. Consequently, the median age of patients in the chemotherapy and non-chemotherapy groups was almost identical (62 vs 63 years, respectively). There were no significant differences between the 2 groups for the features of

*Received 26 October 2000*
*Revised 21 June 2001*
*Accepted 3 July 2001*

*Correspondence to:* B Iacopetta patient sex, tumour site, histological grade, MSI phenotype or *TP53* mutation (results not shown). Information on adjuvant treatment and patient survival was obtained from hospital records and the Health Department of Western Australia. The majority of non-chemotherapy patients were from 1985–1990 whereas all chemotherapy-treated patients were from 1991–1998. Median follow-up time was 115 months for non-chemotherapy-treated patients and 29 months for chemotherapy-treated patients. Only deaths due to recurrence of CRC were considered in the survival analysis. For comparison of TS genotype frequencies, DNA from breast cancer, Dukes' B CRC, and non-cancer patients from the same institute were also analysed.

### TS genotyping

Formalin-fixed, paraffin-embedded surgical tumour specimens were used as the source of DNA for TS genotyping of each patient in the study. The *TS* gene is located on chromosomal arm 18p, a region showing frequent allelic loss in CRC. Because primary CRCs always contain a relatively large proportions of non-tumour tissue (stroma, lymphoid, non-tumour areas) contributing germline DNA, genotyping of the *TS* gene is unlikely to have been affected by the presence of LOH at 18p in the tumour DNA. DNA was extracted following proteinase-K digestion of 20 µm tissue sections as previously described by our laboratory (Soong and Iacopetta, 1997). PCR amplification of the TS promoter enhancer region containing the double and triple tandem repeats was carried out using the following primers:

forward 5′ AAAAGGCGCGCGGAAGGGGTCCT 3′
reverse 5′ TCCGAGCCGGCCACAGGCAT 3′

PCR reactions were carried out in 15 µl volumes comprising 1 µl of DNA preparation, 0.2 U of Taq Polymerase (Qiagen, Australia), primers at a final concentration of 0.4 µM, DMSO at a final concentration of 10%, $Mg^{2+}$ at a final concentration of 3 mM and 5 × reaction mix (Bio Tech International, Perth) containing nucleotides and buffer. A total of 32 PCR cycles were carried out (94°C for 40 s, 62°C for 40 s and 72°C for 1 min) following hot-start at 94°C for 5 min during which time DNA was added. PCR product containing triple repeats (144 bp) was distinguished from that containing double repeats (116 bp) by electrophoretic separation on 3% agarose gels. Patients who were homozygous for the triple repeat (3R/3R) displayed only the larger PCR product, those homozygous for the double repeat (2R/2R) displayed only the smaller PCR product, while heterozygous individuals (2R/3R) showed both the larger and smaller PCR products. All TS genotype results were confirmed at least once by separate PCR reaction.

### Statistical analysis

Associations between TS genotype and various clinico-pathological features of tumours were tested by chi-square analysis. Survival curves were constructed using the method of Kaplan–Meier and compared using the log-rank test. Cox regression analysis was used to estimate the effect of chemotherapy on survival. Statistical significance was assumed when $P < 0.05$ and all analyses were carried out using the SPSS statistical software package.

### RESULTS

TS genotype frequencies for non-cancer, breast cancer and Dukes' B and C CRC patients are compared in Table 1 against frequencies reported by Marsh et al (1999) for a Caucasian population. The 3R/3R homozygote frequency in non-cancer and Dukes' B CRC patients is similar to that reported by Marsh et al, however its frequency in breast carcinoma and Dukes' C CRC patients is significantly lower ($P < 0.05$ and $P < 0.04$, respectively). Since it has been reported that TS levels in tumours from individuals with the 3R/3R genotype are significantly higher than those found in tumours from either 2R/2R or 2R/3R individuals (Kawakami et al, 1999), the latter 2 groups were combined for the purposes of the current analysis. No significant associations between TS genotype and any of the clinicopathological features or genetic alterations were observed (Table 2). A trend for increased frequency of the 3R/3R genotype in patients with poorly differentiated tumours was not confirmed by analysis of larger numbers of well and poorly differentiated tumours (17/60 vs 37/109 respectively, $P$ = NS).

The survival benefit from chemotherapy in the 2 patient groups stratified according to TS genotype is shown in Figure 1. A short-term benefit was apparent for the 3R/3R genotype, but no significant long-term gains from the use of chemotherapy were seen for this patient group ($P$ = 0.18). In contrast, the combined 2R/2R and

**Table 1** TS genotype frequency

| Study (*n*) | 2R/2R | 2R/3R | 3R/3R | $P^a$ |
|---|---|---|---|---|
| Marsh et al (96) | 19% | 43% | 38% | |
| Present study | | | | |
| non-cancer (91) | 22% | 44% | 34% | NS |
| breast cancer (317) | 22% | 51% | 27% | < 0.05 |
| Dukes' B CRC (83) | 17% | 43% | 40% | NS |
| Dukes' C CRC (221) | 28% | 46% | 26% | < 0.04 |

[a]Frequency of the 3R/3R genotype is compared to that reported by Marsh et al (1999).

**Table 2** Association of TS genotype with clinico-pathological features and genetic alterations in Dukes' C CRC

| Feature (*n*) | 2R/2R or 2R/3R (%) | 3R/3R (%) | *P* |
|---|---|---|---|
| Total (221) | 163 (74) | 58 (26) | |
| Male (117) | 88 (75) | 29 (25) | |
| Female (104) | 75 (72) | 29 (28) | 0.60 |
| Left-sided (128) | 93 (73) | 35 (27) | |
| Right-sided (91) | 68 (75) | 23 (25) | 0.73 |
| < 62 years (110) | 81 (74) | 29 (26) | |
| ≥ 62 years (111) | 82 (74) | 29 (26) | 0.97 |
| Well differentiated (22) | 18 (82) | 4 (18) | |
| Moderately differentiated (143) | 108 (76) | 35 (24) | |
| Poorly differentiated (48) | 30 (62) | 18 (38) | 0.13 |
| No chemotherapy (104) | 78 (75) | 26 (25) | |
| Chemotherapy (117) | 85 (73) | 32 (27) | 0.69 |
| MSI– (168) | 119 (71) | 49 (29) | |
| MSI+ (11) | 10 (91) | 1 (9) | 0.15 |
| *TP53* IHC[a] normal (131) | 101 (77) | 30 (23) | |
| *TP53* IHC abnormal (56) | 39 (70) | 17 (30) | 0.28 |
| *TP53* wild-type (137) | 104 (76) | 33 (24) | |
| *TP53* mutation (64) | 44 (69) | 20 (31) | 0.28 |

[a]IHC: immunohistochemical detection of *TP53* overexpression.

 © 2001 Cancer Research Campaign

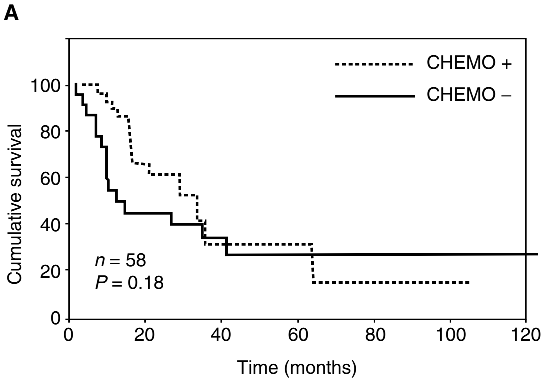

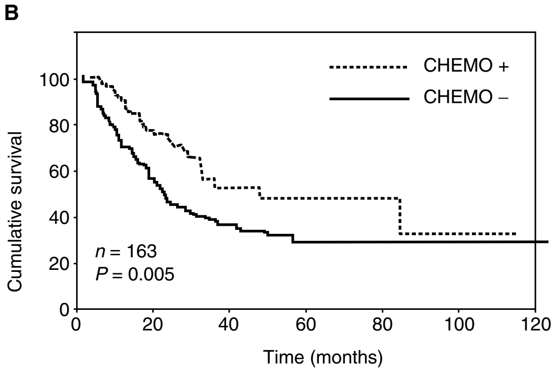

**Figure 1** Kaplan–Meier analysis comparing the survival of adjuvant-treated (chemo+) and non-treated (chemo–) Dukes' C CRC patient groups having (**A**), the 3R/3R TS genotype, (**B**), the 2R/2R or 2R/3R TS genotype

2R/3R patient group showed significant benefit (Figure 1B). Cox regression analysis of the survival benefit conferred by chemotherapy in TS genotype subgroups is shown in Table 3. In chemotherapy-treated patients, the 3R/3R group showed worse survival compared to the combined 2R/2R and 2R/3R group (RR = 1.48, 95% CI: 0.78–2.80, $P = 0.23$).

### DISCUSSION

In several studies of tumour cell lines (Johnston et al, 1992; Beck et al, 1994; van Triest et al, 1999) and clinical samples (Peters et al, 1994; Lenz et al, 1996; Leichman et al, 1997; Salonga et al, 2000), the TS activity level was found to be predictive for response to 5-FU. Most studies report that high tumour TS levels are associated with poor response to chemotherapy, however Johnston et al (1994) found no association with patient survival. Additionally, studies carried out in vitro (Horie et al, 1995) and on gastrointestinal tumours (Kawakami et al, 1999) have shown that the 3R/3R TS genotype is associated with significantly higher TS expression levels compared to the 2R/2R and 2R/3R variants. In the present study we therefore tested the hypothesis that the 3R/3R genotype, because of its links with higher TS levels, is associated with less survival benefit from chemotherapy compared to the 2R/2R and 2R/3R genotypes. We used disease-specific survival as the endpoint in a series of 221 Dukes' C CRC patients, 117 (53%) of whom had been treated post-surgically with 5-FU-based chemotherapy. Although non-randomized, the adjuvant treated and non-treated patient groups were well matched for age, sex, tumour site and histological grade. In line with results from clinical trials (Moertel, 1994), the use of chemotherapy was associated with a significantly reduced risk of death in the overall patient group (Table 3).

**Table 3** Univariate analysis of the effect of chemotherapy on survival of Dukes' C CRC patients with different TS genotypes

| TS genotype | Relative risk[a] | 95% CI | P |
|---|---|---|---|
| Total (221) | 0.56 | 0.38–0.82 | 0.003 |
| 3R/3R (58) | 0.62 | 0.30–1.25 | 0.18 |
| 2R/2R or 2R/3R (163) | 0.52 | 0.33–0.82 | 0.005 |

[a]For each group, the survival of patients treated with chemotherapy is compared to that of patients treated with surgery alone.

The frequency of the 3R/3R genotype found in Dukes' C CRC patients was lower than that observed in this study for non-cancer and Dukes' B CRC patients, as well as that reported recently for Caucasian individuals (Table 1). It is not known whether the apparent differences in TS genotype frequencies between the normal, CRC and breast cancer series shown in Table 1 are real or whether they merely reflect sample size differences. By influencing TS expression levels and therefore the availability of thymidylate, an essential precursor of DNA synthesis, TS genotype could conceivably be an important factor for cancer development and progression. Further studies in large tumour series will be required to address this interesting possibility. The results shown in Table 2 suggest however that TS genotype does not influence the sex, site or age at which CRC develops, nor the histological grade. The trend for association of the 3R/3R genotype with poorly differentiated tumours was not confirmed by analysis of a larger number of cases. The results of our study also suggest that TS genotype does not influence the pathway of CRC tumorigenesis (*TP53* mutant or MSI phenotype), however it should be cautioned that the number of cases examined is small.

The major finding of our work is that CRC patients with the 3R/3R TS genotype derive less survival benefit from chemotherapy than those with the 2R/2R or 2R/3R genotypes (Figure 1 and Table 3). Some short-term benefit was apparent for the 3R/3R patient group and this may be related to the observation by Kawakami et al (1999) that a sizeable fraction of 3R/3R gastrointestinal cancer patients have low tumour TS levels. These patients would be expected to derive benefit from 5-FU-based chemotherapy and may explain the survival benefit seen for the 3R/3R group in the present study. The results of Kawakami et al (1999) demonstrate that TS levels are not tightly linked to TS genotype and other, unidentified factors are likely to play a role in regulating the expression of this gene. The relatively weak association observed between TS genotype and survival benefit from chemotherapy in CRC indicates that direct biochemical or immunohistochemical evaluation of TS level will be required if this predictive marker is to find routine clinical use. Although there are problems with standardization, TS immunohistochemistry has been carried out by many laboratories and it should be possible to distinguish between high- and low-expressing tumours in routine laboratories. It remains to be determined how additional TS alleles containing up to 9 copies of the repeat in some ethnic groups (Marsh et al, 1999) are likely to influence expression levels and therefore the survival benefit from therapy.

A recent study found that advanced CRC patients with low tumour levels of each of the TS, dihydropyrimidine dehydrogenase (DPD) and thymidine phosphorylase (TP) enzymes showed

© 2001 Cancer Research Campaign much greater survival benefit from 5-FU than those with high expression levels (Salonga et al, 2000). The predictive value of such markers should now be tested in prospective trials in conjunction with somatic genetic alterations such as *TP53* mutation (Ahnen et al, 1998) and MSI (Elsaleh et al, 2000) which have also been shown to have strong predictive value. It is tempting to speculate that the recently described methylator phenotype (Toyota et al, 1999) may be relevant to the observations made by Salonga et al (2000). Transcriptional silencing of genes such as *TS*, *TP* and *DPD* by CpG island methylation may be the primary cause for their reduced expression in some tumour tissues. Further prospective studies will be required to uncover both genotype-related and tumour-specific factors that regulate the expression of genes involved in tumour cell response to 5-FU.

### ACKNOWLEDGEMENTS

This work was supported by a grant from the Raine Medical Research Foundation.

### REFERENCES

Ahnen DJ, Feigl P, Quan G, Fenoglio-Preiser C, Lovato LC, Bunn PA et al (1998) Ki-ras mutation and p53 overexpression predict the clinical behavior of colorectal cancer: a Southwest Oncology Group study. *Cancer Res* **58**: 1149–1158

Anonymous (1999) Efficacy of adjuvant fluorouracil and folinic acid in B2 colon cancer: International Multicentre Pooled Analysis of B2 Colon Cancer Trials (IMPACT B2) Investigators. *J Clin Oncol* **17**: 1349–1355

Beck A, Etienne MC, Cheradame S, Fischel JL, Formento P, Renee N et al (1994) A role for dihydropyrimidine dehydrogenase and thymidylate synthase in tumor sensitivity to 5-fluorouracil. *Eur J Cancer* **30**: 1517–1522

Elsaleh H, Joseph D, Grieu F, Zeps N, Spry N and Iacopetta B (2000) Evidence for tumour site and gender specific survival benefit from adjuvant chemotherapy in colorectal cancer. *Lancet* **355**: 1745–1750

Horie N, Hideo A, Oguro K, Hojo H and Takeishi K (1995) Functional analysis and DNA polymorphism of the tandemly repeated sequences in the 5´-terminal regulatory region of the human gene for thymidylate synthase. *Cell Structure Function* **20**: 191–197

Johnston PG, Drake JC, Trepel J and Allegra CJ (1992) Immunological quantitation of thymidylate synthase using the monoclonal antibody TS 106 in 5-fluorouracil-sensitive and resistant human cancer cell lines. *Cancer Res* **52**: 4306–4312

Johnston PG, Fisher ER, Rockette HE, Fisher B, Womark N, Drake JC et al (1994) The role of thymidylate synthase expression in prognosis and outcome of adjuvant chemotherapy in patients with rectal cancer. *J Clin Oncol* **12**: 2640–2647

Kawakami K, Omura K, Kanehira E and Watanabe Y (1999) Polymorphic tandem repeats in the thymidylate synthase gene is associated with its protein expression in human gastrointestinal cancers. *Anticancer Res* **19**: 3249–3252

Leichman CG, Lenz HJ, Leichman L, Danenberg K, Baranda J, Groshen S et al (1997) Quantitation of intratumoral thymidylate synthase expression predicts for disseminated colorectal cancer response and resistance to protracted-infusion fluorouracil and weekly leucovorin. *J Clin Oncol* **15**: 3223–3229

Lenz HJ, Leichman CG, Daneberg KD, Danenberg PC, Groshen S, Cohen H et al (1996) Thymidylate synthase mRNA level in adenocarcinoma of the stomach: a predictor for primary tumor response and overall survival. *J Clin Oncol* **14**: 176–182

Mamounas E, Wieand S, Wolmark N, Bear HD, Atkins JN, Song K et al (1999) Comparative efficacy of adjuvant chemotherapy in patients with Dukes' B versus Dukes' C colon cancer: results from four National Surgical Adjuvant Breast and Bowel Project adjuvant studies (C-01, C-02, C-03, and C-04). *J Clin Oncol* **17**: 1349–1355

Marsh S, Collie-Duguid ESR, Li T, Liu X and McLeod HL (1999) Ethnic variation in the thymidylate synthase enhancer region polymorphism among Caucasian and Asian populations. *Genomics* **58**: 310–312

Moertel CG (1994) Chemotherapy for colorectal cancer. *N Engl J Med* **330**: 1136–1142

Peters GJ, Van der Wilt CL, Van Groeningen CJ, Smid K, Meijer S and Pinedo HM (1994) Thymidylate synthase inhibition after administration of fluorouracil with or without leucovorin in colon cancer patients: implications for treatment with fluorouracil. *J Clin Oncol* **12**: 2035–2042

Salonga D, Danenberg KD, Johnson M, Metzger R, Groshen S, Tsao-Wei DD et al (2000) Colorectal tumors responding to 5-fluorouracil have low gene expression levels of dihydropyrimidine dehydrogenase, thymidylate synthase, and thymidine phosphorylase. *Clin Cancer Res* **6**: 1322–1327

Santi DV, McHenry CS and Sommer H (1974) Mechanism of interaction of thymidylate synthetase with 5-fluorodeoxuridylate. *Biochemistry* **13**: 471–481

Soong R and Iacopetta B (1997) A rapid and non-isotopic method for the screening and sequencing of p53 gene mutations in formalin-fixed, paraffin-embedded tumors. *Modern Pathology* **10**: 252–258

Toyota M, Ahuja N, Ohe-Toyota M, Herman J, Baylin S and Issa J-P (1999) CpG island methylator phenotype in colorectal cancer. *Proc Natl Acad Sci USA* **96**: 8681–8686

van Triest B, Pinedo HM, van Hensbergen Y, Smid K, Telleman F, Schoenmakers PS et al (1999) Thymidylate synthase level as the main predictive parameter for sensitivity to 5-fluorouracil, but not for folate-based thymidylate synthase inhibitors, in 13 nonselected colon cancer cell lines. *Clin Cancer Res* **5**: 643–654

 *© 2001 Cancer Research Campaign*
